# Supplementary material for: FastGT: an alignment-free method for calling common SNVs directly from raw sequencing reads
Source: Sci Rep. 2017 May 31;7:2537. doi: 10.1038/s41598-017-02487-5 (PMC5451431; doi:10.1038/s41598-017-02487-5)
Supplement: Supplementary file 1 — Supplementary Material [file 41598_2017_2487_MOESM1_ESM.pdf]

Supplementary Material

FastGT: an alignment-free method for calling common SNVs directly from raw sequencing reads

Fanny-Dhelia Pajuste[1*], Lauris Kaplinski[1*], Märt Möls[1,2], Tarmo Puurand[1], Maarja Lepamets[1] & Maido Remm[1]

1. DESCRIPTION OF THE DATA STRUCTURE

Adaptive radix tree layout

FastGT uses adaptive radix tree for storing SNV data associated with each k-mer.

k-mers are encoded 2 bits per nucleotide and stored as bitstrings. Branches can thus be split both between and inside nucleotide (A/C going to one branch and G/T to another).
Each branch may have different number of radix bits from 0 (linear path) to 31 (2^{31} leaves or sub-branches), plus up to 53 bits of unique part of bitstring between branches.

Each leaf encodes up to 26 bits of string suffix, plus one 32-bit value associated with given *k*-mer. The tree does not have root branch in strict sense, but instead the first bits (28 by default) are used to select the subtree. Tree leaves are 64-bit integers. Branch nodes are also encoded in single 64-bit integer, plus one additional integer value for each 2^{radix} branches. Each branch value is either leaf or link to next branching node. The schematic layout of the tree is given in Figure S1 and data types in Figure S2.

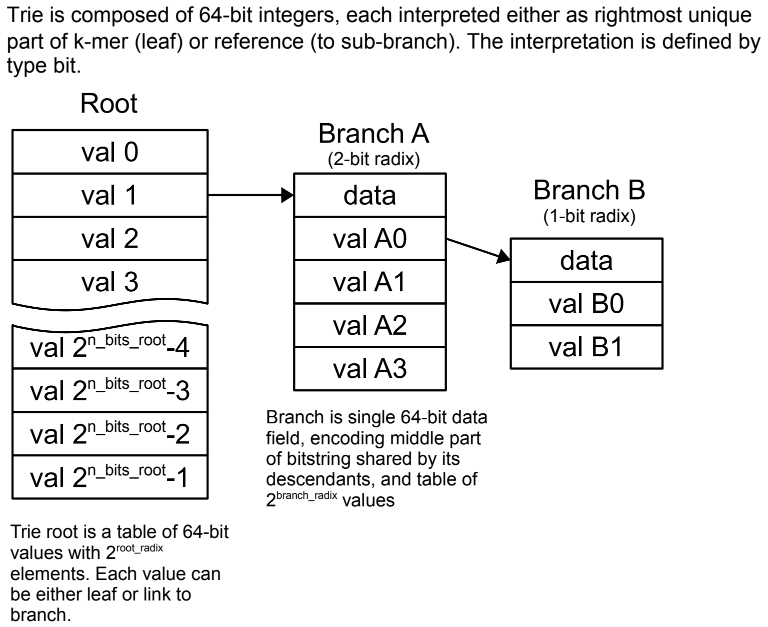

Figure S1. The schematic layout of FastGT *k*-mer tree.

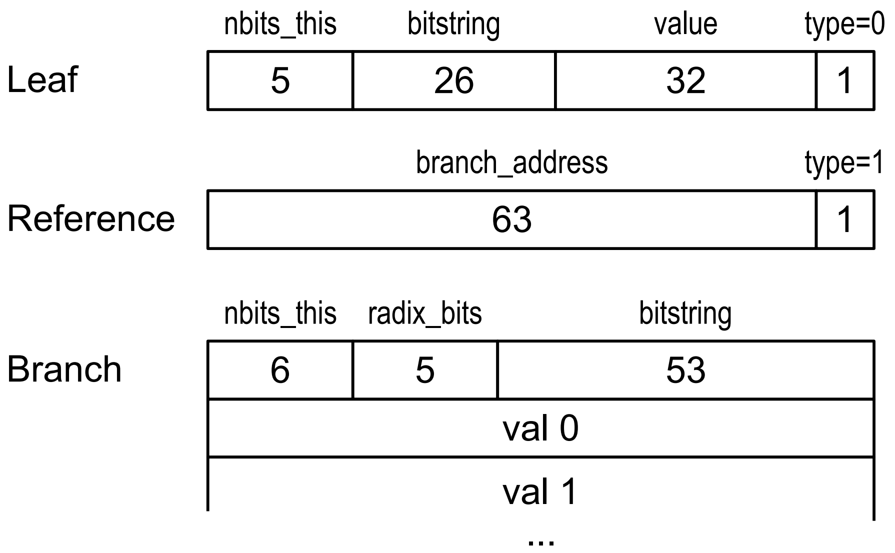

Figure S2. The data types in FastGT *k*-mer tree.

Bitstrings are thus stored as compactly as possible with part of them interpreted as edge indices and parts as values, encoded in edges. An example encoding of 20-mer in tree is shown in Figure S3.

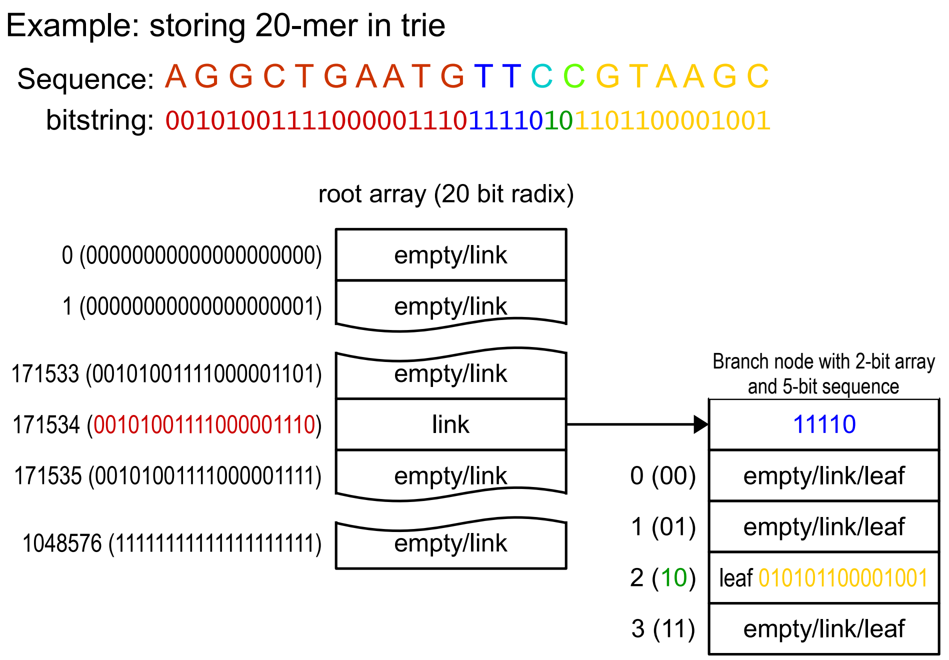

Figure S3. Simplified layout of the tree storing 20-nucleotide (40-bit) k-mer. The initial 20 bits (10 nucleotides, marked with red) form array index into the first subtree node. The next 5 bits (2.5 nucleotides, marked with blue) are encoded in branch data field. The next two bits (spanning 2 nucleotides, marked with green) are used as index into branch descendants (leaves or links to sub-branches). The final 15 bits (7.5 nucleotides, marked with yellow) are encoded in leaf node.

Advantages and dis-advantages of the data structure

The major advantage of trie over hastable-based storage is the inherent ordering. Although not beneficial to FastGT *per se*, the binary *k*-mer trie is meant to be usable in other application where ordering is important - for example set operations between *k*-mer lists.
The variable radix layout allows optimizing database for different types of *k*-mer lookups. For counting of massive number of *k*-mers, large root radix and larger branch radixes for left-side part of *k*-mer should be used. For looking up sparse, semi-random set of *k*-mers, smaller root and binary branches are better suited. FastGT uses fixed initial radix of 28 bits and all-binary branches (1 bit radix).

The main drawback of tree structure in our tests (compared to hash table) is the need of traversing many branches for each *k*-mer lookup, resulting in many potential cache-misses, especially for unsorted lookups. Larger branch radixes will partially compensate for this while introducing trade-off of memory usage by allocating certain number of unoccupied branches. Also inserting unsorted *k*-mers into tree causes adjacent nodes to be spatially separated, resulting in poor cache performance.

The data structure allows parallel modification using multiple threads. For multithreaded insertions blocks of branches and the rootmost radix array are divided between threads.

Performance of the data structure

Lookups are O(log$k$) worst case. Insertions are also O(log$k$) worst case.
The worst-case memory requirement is O(N) in case of binary tree (1-bit radix) split at each bit.

2. STATISTICAL FRAMEWORK

The Empirical Bayes classifier is used to assign the most likely genotype (GT) to each k-mer pair. To calculate the probability of a particular genotype, certain modelling assumptions must be made.

We assume that the k-mer counts C_A and C_B (corresponding to allele A and allele B) for a given genotype have a negative binomial distribution with a mean equal to the product of the coverage (λ) and the true k-mer multiplicity in the genome (the true multiplicity of a A-specific k-mer, denoted by TGC_A, is 2 if the genotype is AA):

$$(C_A|GT, TGC_A \geq 1) \sim NB(mean = TGC_A \cdot \lambda, shape = s_1 + s_2 \cdot TGC_A \cdot \lambda)$$
$$(C_B|GT, TGC_B \geq 1) \sim NB(mean = TGC_B \cdot \lambda, shape = s_1 + s_2 \cdot TGC_B \cdot \lambda),$$

where s_1 and s_2 are unknown parameters estimated from the data.

If the k-mer is not present in the genome ($TGC_A = 0$), then due to sequencing errors there is still a small probability of observing the A-specific k-mer in the sequencing data. If the true multiplicity of a k-mer is 0, we assume that the k-mer count in sequencing reads will have a negative binomial distribution with the parameters

$$(C_A|GT, TGC_A = 0) \sim NB(mean = \lambda_{error} \cdot \lambda, shape = s_1 + s_2 \cdot \lambda_{error} \cdot \lambda)$$
$$(C_B|GT, TGC_B = 0) \sim NB(mean = \lambda_{error} \cdot \lambda, shape = s_1 + s_2 \cdot \lambda_{error} \cdot \lambda),$$

where the parameter λ_{error} describes the frequency of allele-specific k-mers caused by sequencing errors.

Using the negative binomial distribution, the probabilities $P(C_A|GT)$ and $P(C_B|GT)$ can be calculated. To calculate the probability of a particular k-mer pair count, we assume the independence of k-mer counts given the genotype:

$$P(C_A, C_B|GT) = P(C_A|GT) \cdot P(C_B|GT)$$

To calculate the probability of a true genotype given the k-mer counts C_A, C_B, the Bayes formula can be used:

$$P(GT|C_A, C_B) = P(C_A|GT) \cdot P(C_B|GT) \cdot P(GT)/P(C_A, C_B),$$

where $P(C_A, C_B)$ is the probability of observing a particular pair of k-mer counts. The probability $P(C_A, C_B)$ can be calculated by the following formula

$$P(C_A, C_B) = \sum_{GT} P(C_A|GT) \cdot P(C_B|GT) \cdot P(GT).$$

The most probable genotype is called for each single nucleotide variant (SNV) :

$$\text{called genotype} = \arg\max_{GT} P(GT|C_A, C_B).$$

The genotype probabilities $P(GT)$ are calculated as follows:

$$P(GT = AA) = P(A)^2 \cdot P(\text{bi-allelic genotype})$$
$$P(GT = AB) = 2 \cdot P(A) \cdot P(B) \cdot P(\text{bi-allelic genotype})$$
$$P(GT = BB) = P(B)^2 \cdot P(\text{bi-allelic genotype})$$

$$P(GT = A-) = P(A) \cdot P(\text{mono-allelic genotype})$$
$$P(GT = B-) = P(B) \cdot P(\text{mono-allelic genotype})$$

$$P(GT = --) = P(\text{deleted genotype})$$

$$P(GT = AAA) = P(A)^3 \cdot P(\text{tri-allelic genotype})$$
$$P(GT = AAB) = 3 \cdot P(B) \cdot P(A)^2 \cdot P(\text{tri-allelic genotype})$$
$$...,$$

where $P(A)$ is A allele frequency and B allele frequency is denoted by $P(B) := 1 - P(A)$.

For women 8 free parameters are estimated (for calling SNVs both in autosomes and X-chromosome), for men two separate sets of 8 parameters are used for calling SNVs positioned in autosomes and for calling SNVs in sex chromosomes (16 free parameters altogether).

The A allele frequency (over all SNV's) is used to estimate $P(A)$.

The remaining parameters — the probabilities $P(\text{bi-allelic genotype})$, $P(\text{mono-allelic genotype})$, $P(\text{deleted genotype})$, shape parameters s_1 and s_2, coverage λ and the parameter λ_{error} — are estimated from the data using

maximum likelihood method (by numeric optimization). The probability of more than 2 alleles is calculated as

$$1 - P(\text{bi-allelic genotype}) - P(\text{mono-allelic genotype}) - P(\text{deleted genotype}).$$

If the `gmer_caller` is used with default values, non-canonical genotypes are replaced with NC (no call). If the option `--non_canonical` is used, then also non-canonical (tri-allelic etc) genotypes are called. We discourage the calling of non-canonical genotypes because they may not be true deletions or duplications. For example, the deleted genotype may be called due to the deletion of the region of interest or due to a de-novo mutation near the target SNV.

3. SUPPLEMENTARY FIGURES

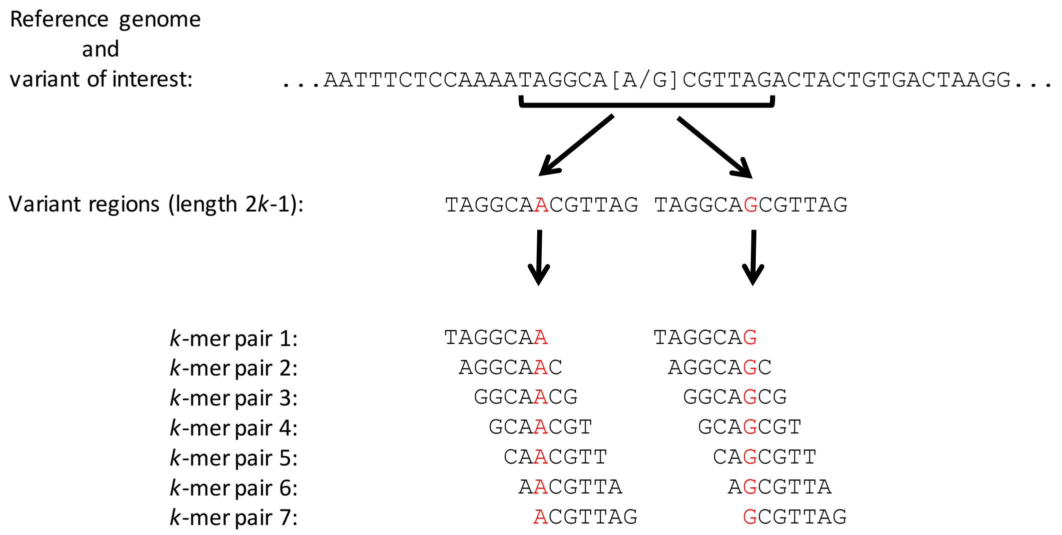

Figure S4. Simplified example of seven *k*-mer pairs (*k*=7) that can be used to distinguish two alleles of an SNV.

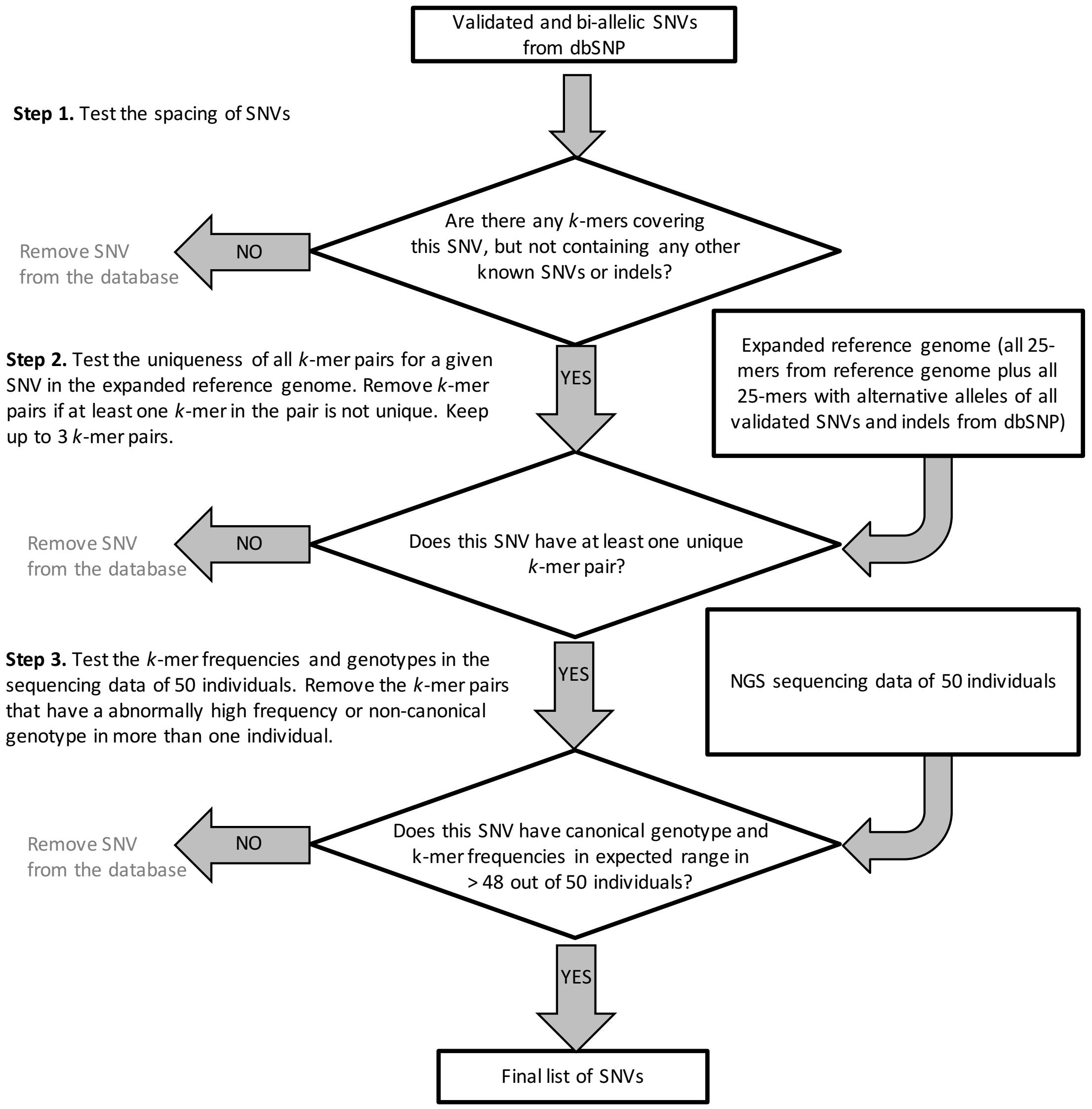

Figure S5. Pipeline for filtering markers.

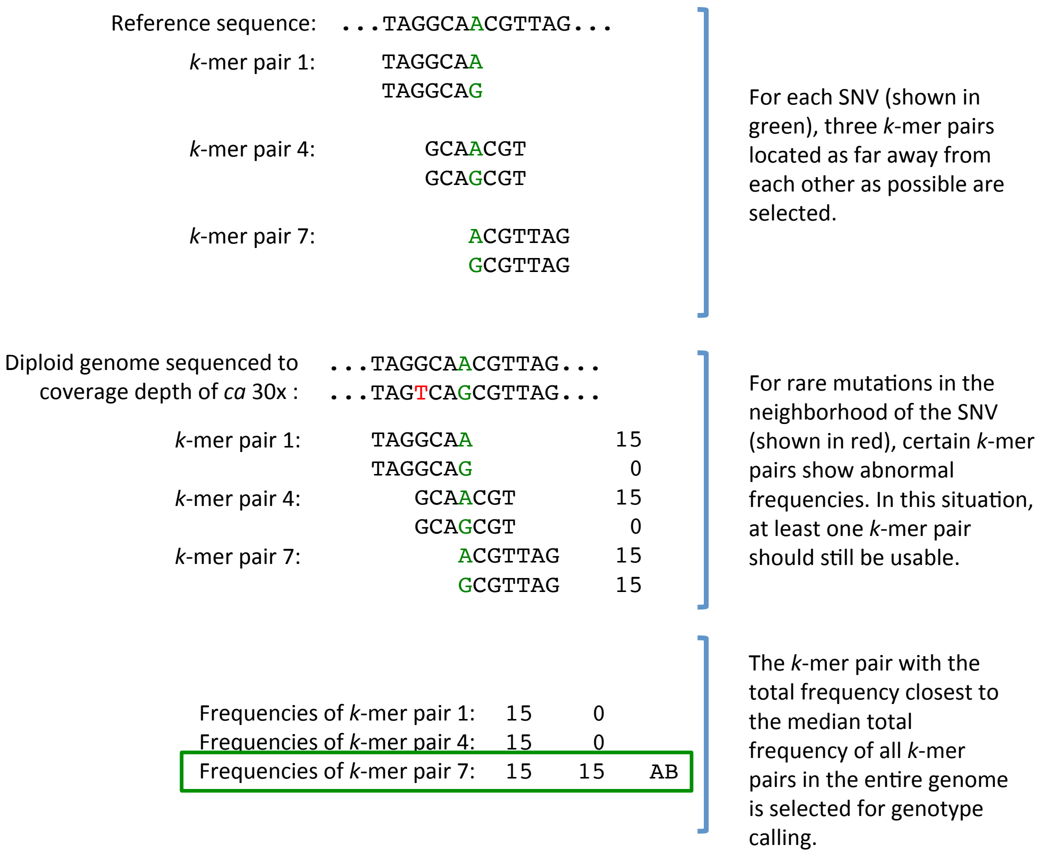

Figure S6. Principles of using redundant *k*-mer pairs for genotyping of a given SNV. Three k-mer pairs that overlap the SNV are selected as follows. Firstly, the attempt is made to select the leftmost pair, the rightmost pair and the pair in the middle of the region. For example, in the case of *k*=7, as shown in this figure, we would prefer to use the 1st, 4th, and 7th *k*-mer pairs. For 25-mers, we prefer to use the 1st, 13th, and 25th *k*-mer pairs. If the most distant *k*-mer pair cannot be used (is not unique or contains SNVs), the next farthest *k*-mer pair is used. The third *k*-mer pair is chosen in the middle at an equal distance from both *k*-mers if possible. Thus, if a rare mutation at one side of the SNV changes the sequence on that side, we expect the *k*-mer pair from the other side to still have the expected counts. Although the frequencies for all three pairs are counted by `gmer_counter`, the genotype calling software `gmer_caller` uses only one pair, which is the pair with a total *k*-mer frequency count that is closest to the median *k*-mer frequency in a given individual.

4. SUPPLEMENTARY TABLES

Table S1. Number and fraction of usable SNVs remaining after subsequent filtering steps.

Dataset	All SNVs from dbSNP	Autosomal SNVs from HumanOmniExpress
Bi-allelic validated SNVs	46,954,719 (100%)	650,307 (100%)
After filtering step 1 (removal of closely located SNVs)	40,946,100 (87%)	596,806 (92%)
After filtering step 2 (removal of SNVs without unique *k*-mer pair)	34,463,965 (73%)	594,762 (91%)
After filtering step 3 (removal of SNVs with abnormal behavior in real data)	30,238,283 (64%)	504,173 (78%)

Table S2. Distribution of all autosomal genotypes inferred by FastGT (rows) from the raw sequencing data of 10 individuals from the Estonian Genome Center and the Illumina HumanOmniExpress microarray genotypes (columns) from the same individuals. The depth of coverage of NGS data in these individuals was between 21 and 35.

		Microarray genotype calls		
		AA	AB	BB
FastGT genotype calls	AA	2,750,130 (54.55%)	1,602 (0.03%)	1,204 (0.02%)
	AB	1,695 (0.03%)	1,477,508 (29.31%)	3,580 (0.07%)
	BB	2 (0.00%)	815 (0.02%)	804,828 (15.96%)
	NC	89 (0.00%)	253 (0.01%)	24 (0.00%)
	concordant (%)	99.94%	99.84%	99.41%

Table S3. Differences in all Y chromosome genotypes inferred by FastGT (rows) and the genotypes in the VCF files of 11 men from the HGDP panel.

		VCF genotype calls		
		AA	AB	BB
FastGT genotype calls	A	247,246 (94.42%)	3,797 (1.45%)	38 (0.01%)
	B	43 (0.02%)	148 (0.06%)	7,446 (2.84%)
	NC	3,026 (1.16%)	82 (0.03%)	41 (0.02%)
	Concordant (%)	99.98%	0%	99.49%
